# Racism and the Life Course: Social and Health Equity for Older Black Americans

**DOI:** 10.1093/geroni/igab046.970

**Published:** 2021-12-17

**Authors:** Linda Chatters, Harry Taylor, Robert Taylor

**Affiliations:** 1 University of Michigan, ANN ARBOR, Michigan, United States; 2 Center for the study of Aging and Human Development, Durham, North Carolina, United States; 3 University of Michigan, Ann Arbor, Michigan, United States

## Abstract

Racism and the Life Course: Social and Health Equity for Older Black Americans examines the impacts of systemic racism on adult development and the aging trajectories of Black Americans. Using the life course perspective (e.g., socio-historical events, linked lives), we discuss systemic racism as a structural driver of practices and policies (e.g., racial residential segregation) that have shaped the social and health circumstances of older Black Americans. These life circumstances include high rates of poverty, poor housing and neighborhood conditions, worse health profiles, and relationship loss and social isolation—conditions that, for too many older Black adults, represent the ‘normal’ state of affairs. Creating a ‘new normal’ of social and health equity for older Black Americans requires recognizing and disrupting the operation of systemic racism in our policies and practices. Selected recommendations and actions for achieving health and social equity for older Black Americans are discussed.

